# Burden and epidemiology of influenza‐ and respiratory syncytial virus‐associated severe acute respiratory illness hospitalization in Madagascar, 2011‐2016

**DOI:** 10.1111/irv.12557

**Published:** 2018-12-27

**Authors:** Joelinotahina H. Rabarison, Stefano Tempia, Aina Harimanana, Julia Guillebaud, Norosoa H. Razanajatovo, Maherisoa Ratsitorahina, Jean‐Michel Heraud

**Affiliations:** ^1^ National Influenza Centre, Virology Unit Institut Pasteur de Madagascar Antananarivo Madagascar; ^2^ Influenza Division National Center for Immunization and Respiratory Diseases US Centers for Disease Control and Prevention Atlanta Georgia; ^3^ Centers for Disease Control and Prevention‐South Africa Pretoria South Africa; ^4^ Centre for Respiratory Diseases and Meningitis National Institute for Communicable Diseases Johannesburg South Africa; ^5^ Epidemiology Unit Institut Pasteur de Madagascar Antananarivo Madagascar; ^6^ Direction de la Veille Sanitaire et de la Surveillance Epidemiologique Ministry of Public Health Antananarivo Madagascar

**Keywords:** Africa, disease burden, hospitalization, influenza, Madagascar, respiratory syncytial virus

## Abstract

**Background:**

Influenza and respiratory syncytial virus (RSV) infections are responsible for substantial global morbidity and mortality in young children and elderly individuals. Estimates of the burden of influenza‐ and RSV‐associated hospitalization are limited in Africa.

**Methods:**

We conducted hospital‐based surveillance for laboratory‐confirmed influenza‐ and RSV‐associated severe acute respiratory illness (SARI) among patients of any age at one hospital and a retrospective review of SARI hospitalizations in five hospitals situated in Antananarivo during 2011‐2016. We estimated age‐specific rates (per 100 000 population) of influenza‐ and RSV‐associated SARI hospitalizations for the Antananarivo region and then extrapolated these rates to the national level.

**Results:**

Overall, the mean annual national number of influenza‐associated SARI hospitalizations for all age groups was 6609 (95% CI: 5381‐7835‐rate: 30.0; 95% CI: 24.4‐35.6), 4468 (95% CI: 3796‐5102‐rate: 127.6; 95% CI: 108.4‐145.7), 2141 (95% CI: 1585‐2734‐rate: 11.6; 95% CI: 8.6‐14.8), and 339 (95% CI: 224‐459‐rate: 50.0; 95% CI: 36.3‐74.4) among individuals aged <5, ≥5, and ≥65 years, respectively. For these same age groups, the mean annual number of RSV‐associated SARI hospitalizations was 11 768 (95% CI: 10 553‐12 997‐rate: 53.4; 95% CI: 47.9‐59.0), 11 299 (95% CI: 10 350‐12 214‐rate: 322.7; 95% CI: 295.6‐348.8), 469 (95% CI: 203‐783‐rate: 2.5;95% CI: 1.1‐4.2), and 36 (95% CI: 0‐84‐rate: 5.8; 0.0‐13.5), respectively.

**Conclusion:**

The burden of influenza‐ and RSV‐associated SARI hospitalization was high among children aged <5 years. These first estimates for Madagascar will enable government to make informed evidence‐based decisions when allocating scarce resources and planning intervention strategies to limit the impact and spread of these viruses.

## 1 INTRODUCTION

Influenza and respiratory syncytial virus (RSV) infections are responsible for substantial global morbidity and mortality annually, with the highest burden experienced by young children and older adults.^1^, ^2^, ^3^, ^4^, ^5^, ^6^, ^7^, ^8^ In addition, a higher burden of influenza‐ and RSV‐associated hospitalization has been reported among African children compared with children in other Regions.^2^, ^5^, ^8^, ^9^ Nonetheless, studies were limited to a pediatric population and estimates from Africa were obtained from a very limited number of participating countries. Estimates of the national burden of influenza‐ and RSV‐associated hospitalization across age groups are severely limited in many African countries, having been described only in five countries for influenza,^3^, ^10^, ^11^, ^12^, ^13^ and two countries for RSV^4^, ^7^, ^10^ on the continent.

The World Health Organization (WHO) has highlighted the need for influenza disease burden estimates especially from low‐ and middle‐income countries. These estimates would enable governments to make informed evidence‐based decisions when allocating scarce resources and planning intervention strategies to limit the impact and spread of the disease.^14^ The effectiveness of RSV candidate vaccines is being evaluated,^15^ and a better understanding of the burden of RSV‐associated illness would assist in the formulation of polices should a vaccine become available.

We aimed to estimate the national and provincial number and rate of influenza‐ and RSV‐associated severe acute respiratory illness (SARI) hospitalizations among patients of different age groups in Madagascar from January 2011 through December 2016. In addition, we compared the epidemiological and clinical characteristics of influenza‐ and RSV‐positive patients hospitalized with SARI.

## 2 METHODS

### 2.1 Data sources

#### 2.1.1 DS‐1: Number of SARI hospitalizations in Antananarivo Renivohitra District

Antananarivo Renivohitra District is situated in the Analamanga Region within Antananarivo Province where the capital city of the country is located. We obtained the total number of patients hospitalized with SARI in Antananarivo Renivohitra District during January 2011 through December 2016 through an anonymized retrospective record review implemented in three public university hospitals (Joseph Raseta Befelatanana hospital, Tsaralalana mother and child hospital, and Ambohimiandra mother and child hospital), one military hospital (Centre Hospitalier Universitaire de Soavinandrina (CENHOSOA)), and one private hospital (Saint‐François d'Assise Ankadifotsy hospital). These hospitals were selected after a pre‐survey conducted to identify all health care facilities within the study district with the capacity of admitting patients with SARI. Six specialty hospitals such as eye or surgical hospitals were excluded from the pre‐survey. Hospital admission books and medical records were reviewed to identify patients hospitalized with signs and symptoms consistent with the WHO SARI case definition reported below. For each identified patient admitted at the selected hospitals, age, gender, dates of admission and discharge, and location of residence were recorded.

#### 2.1.2 DS‐2: Influenza and RSV surveillance among patients hospitalized with SARI

We conducted active, prospective, hospital‐based surveillance for SARI at the CENHOSOA from January 2011 through December 2016. Surveillance activities were conducted at the pediatric and adult pneumology wards. A SARI patient was defined as a hospitalized person of any age presenting with (a) either a recorded temperature ≥38°C or history of fever and (b) cough of duration of ≤10 days.^14^ Trained surveillance nurses completed case report forms that included demographic, clinical, and epidemiological information for all SARI cases. In addition, respiratory specimens (nasopharyngeal and oropharyngeal swabs) were collected from all consenting patients. Specimens were placed in vials containing universal transport medium, stored at 4‐8°C, and transported to the National Influenza Center (located at the Institute Pasteur de Madagascar) within 24 hours of collection for testing. Specimens were tested within 48 hours from collection for influenza A and B viruses and RSV using a real‐time reverse transcription polymerase chain reaction assay.^16^, ^17^ Influenza A‐positive samples were further subtyped. All patients meeting the SARI case definition were eligible for enrollment. Verbal informed consent was obtained from all patients prior to data and specimen collection. For children <15 years, verbal consent was obtained from a parent or legal guardian.

#### 2.1.3 DS‐3: Prevalence of risk factors for pneumonia and health care seeking behavior for acute respiratory infection

We obtained the regional‐level prevalence (22 regions) of known risk factors for pneumonia and regional data on health care seeking behavior among individuals with acute respiratory infection (ARI) from the 2008‐2009 Madagascar Demographic and Health Survey (DHS).^18^

#### 2.1.4 DS‐4: Population denominators

District, region, and province age‐ and year‐specific population denominators were obtained from projections of the 1993 census data for Madagascar.^19^ Madagascar had an estimated population of 23 719 450 individuals in 2016 of which 3 771 450 (15.9%) were children aged <5 years.

### 2.2 Estimation of the national number and rate of influenza‐ and RSV‐associated SARI hospitalization

**Figure 1:**
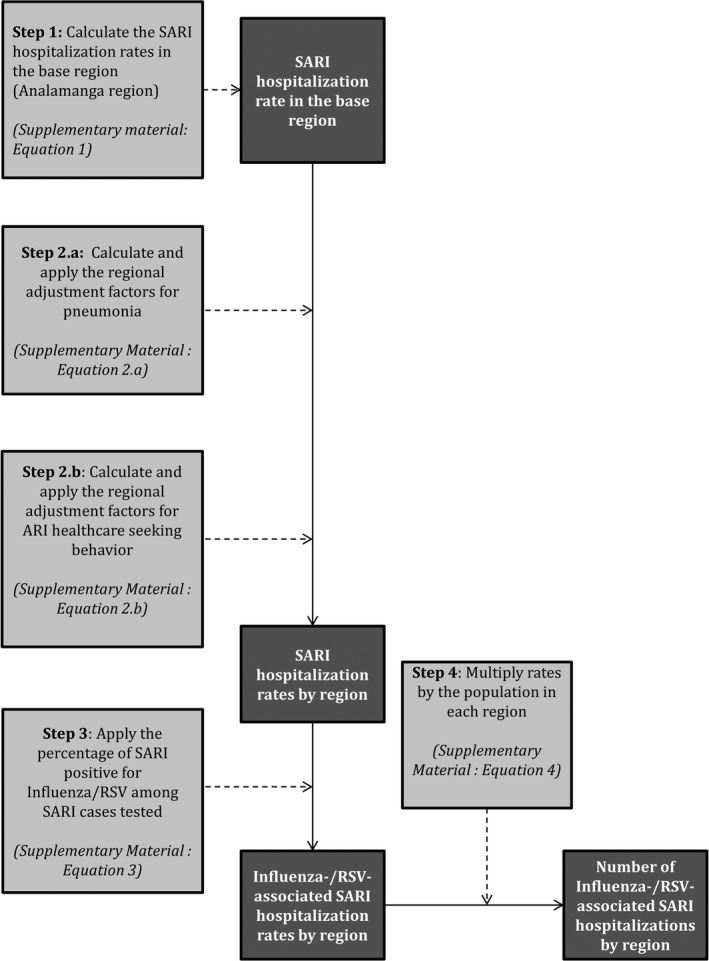
Method used to estimate the numbers and rates of influenza‐ and respiratory syncytial virus‐associated severe acute respiratory illness hospitalization in Madagascar, 2011‐2016. Data inputs steps are in light gray boxes, and data outputs are in dark gray boxes

To estimate the national number and rate of influenza‐ and RSV‐associated SARI hospitalization, we used a four‐step approach. In Step 1, we estimated the SARI hospitalization rate in Analamanga Region considered to be the base region in our estimation approach (where we conducted the retrospective record review and the prospective hospital‐based surveillance for SARI). In Step 2, we estimated the SARI hospitalizations rates for the other regions using estimates from the base region. In Step 3, we estimated the influenza‐ and RSV‐associated SARI hospitalizations rates using available virologic surveillance data for influenza and RSV. In Step 4, we obtained the number of influenza‐ and RSV‐associated SARI hospitalizations using the estimated rates and the population at risk in each region.^13^, ^20^, ^21^ The description of the estimation approach for each step is provided below and in Figure 1. The equations used for the estimations are provided in the Methods section of the Supporting Information. All estimates were obtained overall and within the following age categories: <5, 5‐24, 25‐44, 45‐64, ≥65, and ≥5 years of age. Rates were expressed per 100 000 population. All estimates were reported as mean annual estimates over the study period. Annual estimates were also provided overall and among individuals aged <5 and ≥5 years.

#### 2.2.1 Step 1: Estimation of SARI hospitalizations rates in Analamanga region

To estimate the SARI hospitalizations rates in Analamanga Region, we followed the WHO Manual for Estimating the Disease Burden Associated with Seasonal Influenza.^14^ First, we obtained the SARI hospitalization rates for the Antananarivo Renivohitra District by dividing the total number of SARI hospitalizations that occurred in the district (DS‐1) by the district population (DS‐4). Thereafter, we used the Antananarivo Renivohitra District SARI hospitalization rates as a proxy for Analamanga Region as previously described.^12^, ^13^, ^20^, ^21^

#### 2.2.2 Step 2: Estimation of SARI hospitalizations rates in the other regions

Estimates of SARI hospitalization rates for the other 21 regions in Madagascar were derived by adjusting the Analamanga Region rate (base region—obtained in Step 1) for the regional‐level prevalence of known risk factors for pneumonia obtained from the DHS (DS‐3) as previously described (Step 2.a).^13^, ^20^, ^21^ Risk factors included exposure to indoor air pollution, crowding, malnutrition, low birthweight, and non‐exclusive breastfeeding.^13^, ^20^, ^21^ The last three were only included for children aged <5 years. HIV infection prevalence, which was used as an adjustment factor in other similar studies, was excluded in our analysis because of the very low prevalence of HIV infection in Madagascar (<0.2% in the general population)^22^ and the lack of region‐specific prevalence estimates. The relative risk of SARI associated with each risk factor was determined from the published literature.^13^, ^20^, ^21^, ^23^ In addition, we adjusted the regional rates by the proportion of ARI cases seeking care in the given region to the proportion of ARI cases seeking care in the base region using data from the DHS (DS‐3) as previously described (Step 2.b).^13^, ^20^, ^21^ The differential health care seeking behavior between regions among patients with ARI was used as a proxy for the differential health care seeking behavior among patients with SARI. An adjustment factor >1 resulted in a greater SARI hospitalization rate in the given region relative to the base region and vice versa. The equations used for the regional adjustments are provided in the Supporting Information.

#### 2.2.3 Step 3: Estimation of influenza‐ and RSV‐associated SARI hospitalizations rates in all regions

We estimated the regional rates of influenza‐ and RSV‐associated SARI hospitalization by multiplying the estimated regional SARI hospitalization rates (obtained in Steps 1 and 2) by the influenza and RSV positivity proportion obtained from influenza and RSV sentinel surveillance implemented among inpatients with SARI (DS‐2).^13^, ^20^, ^21^ The influenza and RSV positivity proportion was the number of positives cases divided by the total number of SARI cases tested.

#### 2.2.4 Step 4: Estimation of the number of influenza‐ and RSV‐associated SARI hospitalizations in all regions

We estimated the regional number of influenza‐ and RSV‐associated SARI hospitalizations by multiplying the regional influenza‐ and RSV‐associated SARI hospitalization rates (obtained in Step 3) by the mid‐year population at risk in each region over the study period.^13^, ^20^, ^21^ We obtained the 95% confidence intervals (CI) using bootstrap resampling over 1000 replications for all parameters included in the calculations.^13^, ^20^, ^21^ This included (a) the age‐ and year‐specific SARI hospitalization rates in the base region; (b) the regional prevalence of the risk factors for pneumonia; (c) the regional proportion of ARI cases seeking care; and (d) the age‐specific influenza and RSV positivity proportion among SARI cases tested. The lower and upper limits of the 95% CI were the 2.5th and 97.5th percentiles of the estimated values obtained from the 1000 resampled datasets, respectively. Finally, we aggregated the regional estimates by the 6 provinces of Madagascar and nationally.

### 2.3 Comparison of the demographic and clinical characteristics of influenza‐ and RSV‐positive patients hospitalized with SARI

We compared the demographic and clinical characteristics of RSV‐positive patients to those of influenza‐positive patients hospitalized with SARI (DS‐2) using unconditional logistic regression. For the multivariable model, we assessed all variables that were significant at *P* < 0.2 on univariate analysis and dropped non‐significant factors (*P* ≥ 0.05) with manual backward elimination. Pairwise interactions were assessed by inclusion of product terms for all variables remaining in the final multivariable additive model. Patients in whom influenza virus and RSV were codetected were excluded from this analysis. In addition, for this analysis, we excluded fever and cough as these symptoms were surveillance inclusion criteria.

All statistical analyses were implemented using Stata 14.2 (StataCorp, College Station, TX, USA).

### 2.4 Ethics

The influenza and RSV virologic data (DS‐2) and the collection of SARI hospitalizations data (DS‐1) are part of the disease surveillance system of the Ministry of Public Health (MoH) of Madagascar and were deemed non‐research by the Malagasy MoH and the US Centers for Disease Control and Prevention. The retrospective record review (DS‐1) was approved by National Ethic Committee, protocol number 038‐MSANP/CE. The DHS (DS‐3) and census data (DS‐4) were publicly available.

## 3 RESULTS

### 3.1 Number of SARI hospitalizations in Antananarivo Renivohitra District

During 2011‐2016, there were 12 672 patients hospitalized with SARI recorded from the retrospective record review implemented at the 5 selected hospitals of which 4650 (36.4%) resided outside Antananarivo Renivohitra District and were excluded from further analysis (DS‐1). Of those not residing in the study district, 4203 (90.4%) resided in neighboring Districts located within Analamanga Region and the remaining in adjacent Regions. Of the 8022 patients hospitalized with SARI residing in Antananarivo Renivohitra District, 6170 (76.9%) were children aged <5 years.

### 3.2 Influenza and RSV surveillance among patients hospitalized with SARI

**Figure 2:**
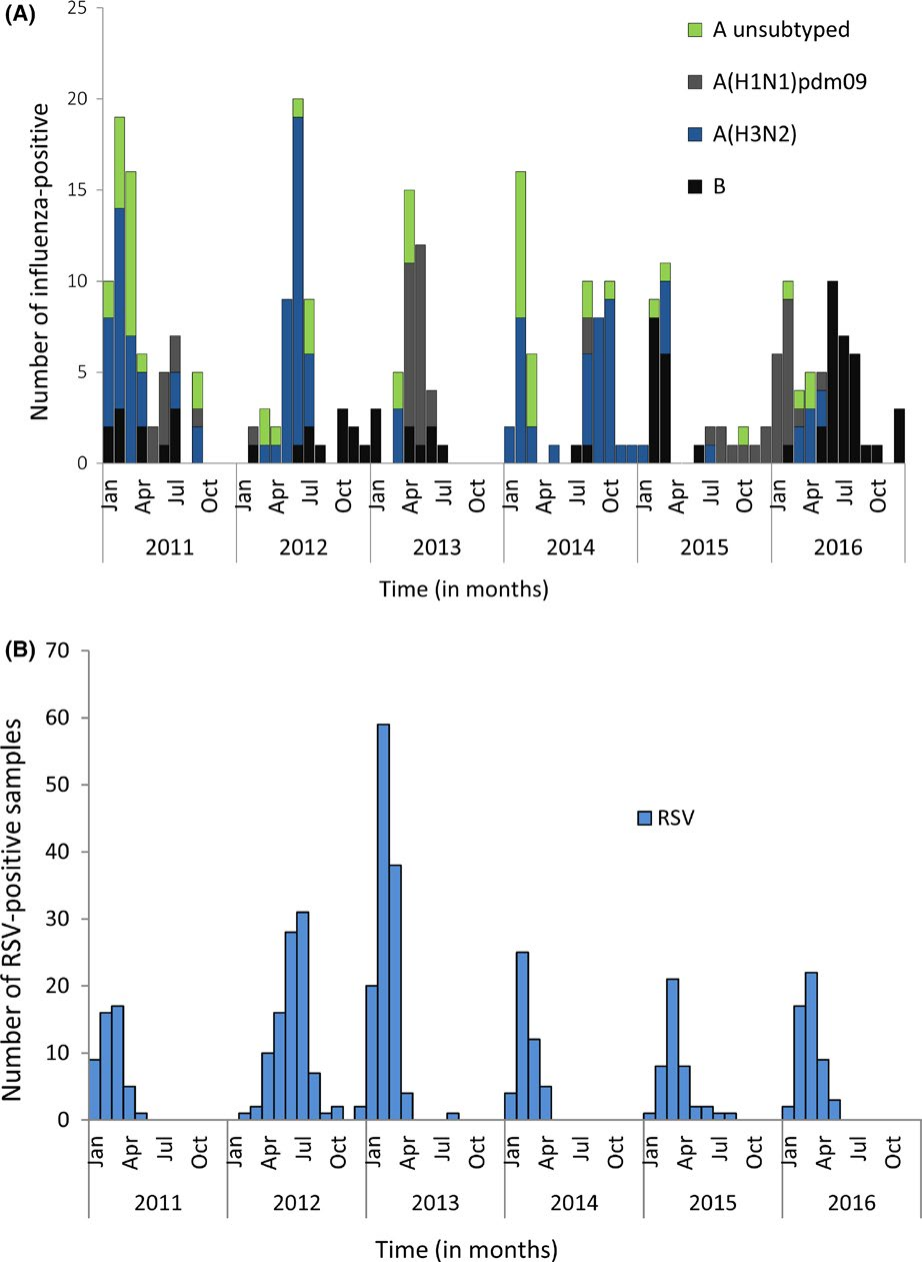
Monthly number of influenza‐ and respiratory syncytial virus‐positive cases among patients hospitalized with severe acute respiratory illness at the Centre Hospitalier de Soavinandrina, Analamanga Region, Madagascar, 2011‐2016. A, Influenza virus; B, respiratory syncytial virus

During 2011‐2016, we identified 2511 patients hospitalized with SARI at the CENHOSOA during the retrospective record review (DS‐3). Of these patients, 1323 (52.7%) were enrolled for virologic surveillance of which 799 (60.4%) were children aged <5 years (DS‐2). Overall influenza viruses were detected in 23.3% (308/1323) of specimens, in 18.9% (151/799) and 30.0% (157/524) of specimens among individuals aged <5 and ≥5 years, respectively. Of the 308 influenza‐positive specimens, 117 (38.0%) were influenza A(H3N2), 58 (18.8%) were influenza A(H1N1)pdm09, 54 (17.5%) were influenza A not subtyped, and 79 (25.7%) were influenza B viruses. Influenza virus was detected throughout the year with peak circulation periods observed during January‐April and June‐August (Figure 2A). Overall, RSV was detected in 31.2% (413/1323) of specimens, in 47.7% (381/799) and 6.1% (32/524) of specimens among individuals aged <5 and ≥5 years, respectively. RSV was detected predominately during January‐June (Figure 2B).

### 3.3 National number and rates of influenza‐ and RSV‐associated SARI hospitalization

In individuals of all ages, the estimated mean annual number of influenza‐associated SARI hospitalization was 6609 (95% CI: 5381‐7835; rate: 30.0, 95% CI: 24.4‐35.6 per 100 000 population), 4468 (67.6%) (95% CI: 3796‐5102; rate: 127.6, 95% CI: 108.4‐145.7 per 100 000 population), and 2141 (32.4%) (95% CI: 1585‐2734; rate: 11.6, 95% CI: 8.6‐14.8 per 100 000 population) among individuals aged <5 and ≥5 years, respectively (Table 1). The estimated mean annual rates of influenza‐associated SARI hospitalization were highest among children aged <5 year and lowest among individuals aged 25‐44 years (rate: 7.6, 95% CI: 5.5‐10.2 per 100 000 populations).

**Table 1:** Estimated mean annual number and rate of influenza‐ and respiratory syncytial virus‐associated severe acute respiratory illness hospitalization, Madagascar, 2011‐2016

Age group (in y)	Influenza‐associated SARI hospitalization		RSV‐associated SARI hospitalization	
	Number (95% CI)	Rate (95% CI)^a^	Number (95% CI)	Rate (95% CI)^a^
<5	4468 (3796‐5102)	127.6 (108.4‐145.7)	11 299 (10 350‐12 214)	322.7 (295.6‐348.8)
5‐24	949 (730‐1175)	9.1 (7.0‐11.3)	282 (147‐431)	2.7 (1.4‐4.1)
25‐44	395 (286‐531)	7.6 (5.5‐10.2)	70 (23‐132)	1.3 (0.4‐2.5)
45‐64	458 (346‐570)	20.0 (15.1‐24.9)	81 (34‐137)	3.5 (1.5‐6.0)
≥65	339 (224‐459)	55.0 (36.3‐74.4)	36 (0‐84)	5.8 (0.0‐13.5)
≥5	2141 (1585‐2734)	11.6 (8.6‐14.8)	469 (203‐783)	2.5 (1.1‐4.2)
All	6609 (5381‐7835)	30.0 (24.4‐35.6)	11 768 (10 553‐12 997)	53.4 (47.9‐59.0)
Province				
Antananarivo	2114 (1721‐2497)	32.8 (26.7‐38.8)	3734 (3367‐4109)	58.0 (52.3‐63.8)
Antsiranana	373 (303‐440)	21.9 (17.7‐25.8)	660 (588‐731)	38.7 (34.5‐42.8)
Fianarantsoa	1362 (1111‐1619)	29.5 (24.1‐35.1)	2464 (2204‐2723)	53.4 (47.8‐59.1)
Mahajanga	735 (597‐874)	27.5 (22.3‐32.7)	1264 (1128‐1406)	47.4 (42.3‐52.7)
Toamasina	1033 (845‐1227)	32.0 (26.1‐38.0)	1884 (1689‐2083)	58.3 (52.2‐64.4)
Toliara	992 (806‐1180)	29.5 (24.0‐35.1)	1762 (1577‐1947)	52.4 (46.9‐57.9)

SARI, severe acute respiratory illness; RSV, respiratory syncytial virus; CI, confidence intervals.

a Rates expressed per 100 000 population.

In individuals of all ages, the estimated mean annual number of RSV‐associated SARI hospitalization was 11 768 (95% CI: 10 553‐12 997; rate: 53.4, 95% CI: 47.9‐59.0 per 100 000 population), 11 299 (96.0%) (95% CI: 10 350‐12 214; rate: 322.7, 95% CI: 295.6‐348.8 per 100 000 population), and 469 (4.0%) (95% CI: 203‐783; rate: 2.5, 95% CI: 1.1‐4.2 per 100 000 population) among individuals aged <5 and ≥5 years, respectively (Table 1). The estimated mean annual rate of RSV‐associated SARI hospitalization was highest among children aged <5 years and lowest among individuals aged 25‐44 years (rate: 1.3, 95% CI: 0.4‐2.5 per 100 000 population).

A U‐shaped trend of the magnitude of the mean annual influenza‐ and RSV‐associated SARI hospitalizations rates was observed across age groups (Table 1). Compared to RSV, mean annual influenza‐associated SARI hospitalization rates were lower (with non‐overlapping CIs) among children aged <5 years and higher among individuals aged ≥5 years. No substantial differences (with overlapping CIs) of the influenza‐ and RSV‐associated SARI hospitalizations rates were observed between provinces (Table 1); however, variations were observed between years (Table 2). The provincial mean annual number and rates of influenza‐ and RSV‐associated SARI hospitalizations by age group are provided in Table S1. The influenza‐ and RSV‐associated SARI hospitalization rates by region are provided in Figure S1.

**Table 2:** Estimated annual number and rate of influenza‐ and respiratory syncytial virus‐associated severe acute respiratory illness hospitalization, Madagascar, 2011‐2016

Age group (in y)	Influenza‐associated SARI hospitalization		RSV‐associated SARI hospitalization	
	Number (95% CI)	Rate (95% CI)^a^	Number (95% CI)	Rate (95% CI)^a^
2011				
<5	7243 (5325‐9141)	220.1 (161.8‐277.8)	8003 (6105‐10 028)	243.2 (185.5‐304.7)
≥5	4083 (2190‐6194)	23.5 (12.6‐35.6)	777 (0‐2171)	4.5 (0.0‐12.5)
All	11 326 (7515‐15 334)	54.7 (36.3‐74.1)	8780 (6105‐12 199)	42.4 (29.5‐58.9)
2012				
<5	3726 (2649‐4902)	110.3 (78.4‐145.1)	10 629 (9043‐12 380)	314.7 (267.7‐366.5)
≥5	2013 (563‐3630)	11.3 (3.2‐20.3)	248 (0‐712)	1.4 (0.0‐4.0)
All	5739 (3212‐8531)	27.0 (15.1‐40.2)	10 877 (9043‐13 092)	51.2 (42.6‐61.6)
2013				
<5	2958 (1688‐4196)	88.8 (50.7‐126.0)	18 061 (15 967‐20 138)	542.4 (479.5‐604.8)
≥5	3151 (1061‐5340)	17.9 (6.0‐30.3)	1359 (278‐2707)	7.7 (1.6‐15.4)
All	6109 (2749‐9536)	29.2 (13.1‐45.5)	19 420 (16 245‐22 844)	92.7 (77.6‐109.1)
2014				
<5	9214 (6530‐11 995)	258.3 (183.1‐336.3)	12 832 (10 058‐15 736)	359.7 (282.0‐441.1)
≥5	2080 (901‐3339)	11.0 (4.8‐17.7)	531 (0‐1218)	2.8 (0.0‐6.5)
All	11 294 (7431‐15 334)	50.3 (33.1‐68.3)	13 363 (10 058‐16 954)	59.6 (44.8‐75.6)
2015				
<5	1589 (702‐2638)	43.4 (19.2‐72.0)	6893 (5193‐8839)	188.2 (141.8‐241.4)
≥5	1049 (245‐1965)	5.4 (1.3‐10.1)	362 (0‐808)	1.9 (0.0‐4.2)
All	2638 (947‐4603)	11.5 (4.1‐20.0)	7255 (5193‐9646)	31.5 (22.5‐41.9)
2016				
<5	5002 (3307‐6758)	132.6 (87.7‐179.2)	10 656 (8507‐13 071)	282.5 (225.5‐346.6)
≥5	2049 (950‐3225)	10.3 (4.8‐16.2)	266 (0‐753)	1.3 (0.0‐3.8)
All	7051 (4257‐9982)	29.7 (17.9‐42.1)	10 922 (8507‐13 824)	46.0 (35.9‐58.3)

SARI, severe acute respiratory illness; RSV, respiratory syncytial virus; CI, confidence intervals.

a Rates expressed per 100 000 population.

### 3.4 Comparison of the demographic and clinical characteristics of influenza‐ and RSV‐positive patients hospitalized with SARI

During 2011‐2016, there were 239 patients hospitalized with SARI that tested positive for influenza virus only, 344 that tested positive for RSV only, and 69 that tested positive for both viruses; the latter were excluded for this analysis. On multivariable analysis, compared to individuals that tested positive for influenza virus, those who tested positive for RSV were less likely to be older [5‐24 years (adjusted odds ratio [aOR]: 0.07; 95% confidence interval [CI]: 0.03‐0.15), 25‐44 years (aOR: 0.04; 95% CI: 0.02‐0.11), 45‐64 years (aOR: 0.03; 95% CI: 0.01‐0.08), ≥65+ years (aOR: 0.03; 95% CI: 0.01‐0.09) compared to <5 years of age] but were more likely to have longer duration of hospitalization (aOR: 1.3; 95% CI: 1.1‐1.6) (Table 3).

**Table 3:** Factors associated with respiratory syncytial virus positivity compared to influenza virus positivity among patients hospitalized with severe acute respiratory illness at the Centre Hospitalier de Soavinandrina, Analamanga Region, Madagascar, 2011‐2016

Characteristics	FLU‐positive n/N (%)	RSV‐positive n/N (%)	Univariate analysis		Multivariable analysis	
			OR		Adjusted OR	
	N = 239	N = 344	(95% CI)	*P* a	(95% CI)	*P*
Age (in y)	n (n/N)	n (n/N)				
<5	90 (37.7)	321 (93.3)	*Reference*	–	*Reference*	–
5‐24	41 (17.2)	10 (2.9)	0.07 (0.03‐0.14)	*<0.001*	0.07 (0.03‐0.15)	*<0.001*
25‐44	33 (13.8)	5 (1.5)	0.04 (0.02‐0.11)	*<0.001*	0.04 (0.02‐0.11)	*<0.001*
45‐64	47 (19.7)	5 (1.5)	0.02 (0.01‐0.08)	*<0.001*	0.03 (0.01‐0.08)	*<0.001*
≥65	28 (11.7)	3 (0.9)	0.03 (0.01‐0.10)	*<0.001*	0.03 (0.01‐0.09)	*<0.001*
Gender	n (n/N)	n (n/N)				
Male	132 (55.2)	201 (58.4)	*Reference*	–		
Female	107 (44.8)	143 (41.6)	1.1 (0.8‐1.6)	0.44		
Length of hospitalization	n (n/N)	n (n/N)				
<7 d	78 (32.6)	159 (46.2)	*Reference*	–	*Reference*	–
≥7 d	161 (67.4)	185 (53.8)	1.4 (1.2‐1.6)	*<0.001*	1.3 (1.1‐1.6)	0.037
Clinical signs	n (n/N)	n (n/N)				
Dyspnea	201 (84.1)	302 (87.8)	1.4 (0.8‐2.2)	0.204		
Rhinorrhea	67 (28.0)	155 (45.1)	2.1 (1.5‐3.0)	*<0.001*		
Sore throat	26 (10.9)	22 (6.4)	0.6 (0.3‐1.0)	0.055		
Myalgia	35 (14.6)	4 (1.2)	0.07 (0.02‐0.20)	*<0.001*		
Asthenia	65 (27.2)	76 (22.1)	0.8 (0.5‐1.1)	0.158		

RSV, respiratory syncytial virus; OR, odds ratio; CI, confidence intervals.

a Significant *P*‐values are indicated in italic.

## 4 DISCUSSION

We reported national and provincial estimates of influenza‐ and RSV‐associated SARI hospitalization in Madagascar over a 6‐year period. Influenza‐ and RSV‐associated SARI hospitalizations were substantial with those associated with RSV infection being the highest. Whereas SARI hospitalizations associated with both pathogens were observed across age groups, a differential burden among patients of different age was observed with children aged <5 years accounting for 67.6% and 96.0% of the total number of SARI hospitalizations associated with influenza virus and RSV infections, respectively. For both pathogens, the highest SARI hospitalization rates were observed among children aged <5 years and individuals aged ≥65 years, whereas individuals aged 25‐44 years experienced the lowest rates.

Higher rates of influenza‐associated respiratory hospitalizations among young children and the elderly have been reported in other case‐based and ecological studies.^3^, ^24^ The estimated rates of influenza‐associated SARI hospitalization among Malagasy children aged <5 years (127.6 (108.4‐145.7) per 100 000 population) were similar to those reported from studies conducted in South Africa (range: 153‐186 per 100 000 population),^3^ Kenya (270 per 100 000 population),^25^ Ghana (135 per 100 000 population),^11^ Rwanda (168 per 100 000 population),^12^ and Zambia (187 per 100 000 population)^13^ and global estimates for Africa (174 per 100 000 population),^5^ and such estimates were higher compared to those of other Regions.^5^

Among individuals aged ≥5 years, the Madagascar estimates (11.6 per 100 000 population) were generally consistent with the estimates obtained from similar studies conducted in Africa: South Africa (22.1 per 100 000 population),^3^ Kenya (30.0 per 100 000 population),^25^ Rwanda (11 per 100 000 population),^12^ and Zambia (13 per 100 000 population).^13^ Underlying medical conditions including HIV infection are known risk factors for influenza‐associated severe illness.^3^, ^26^, ^27^, ^28^ Differences in the prevalence of such conditions in these settings may explain some of the observed variation in the influenza‐associated SARI hospitalization rates in this age group. Cultural differences and differential access to health care across countries can also play a role in health care seeking behavior potentially also contributing to variability in hospitalization rates.

The estimated rates of RSV‐associated SARI hospitalization among individuals aged <5 years (322.7 per 100 000 population) and ≥5 years (2.5 per 100 000 population) in Madagascar were lower than those reported in South Africa (1000 per 100 000 population among children aged <5 years vs 30 per 100 000 population among individuals aged ≥5 years).^4^, ^7^ Kenya studies reported RSV‐associated SARI hospitalization rates of 1360 and 11 per 100 000 population among patients aged <5 and ≥5 years, respectively.^10^ In a global study of RSV‐associated respiratory hospitalization among children aged <5 years, rates of 560 per 100 000 population were reported for low‐income countries^8^; however, in population‐based studies conducted in low‐ to high‐income countries across the world, the RSV‐associated hospitalization rate among individuals aged >5 years ranged from 2.9 to 130 per 100 000 population.^29^, ^30^, ^31^, ^32^, ^33^, ^34^, ^35^

SARI patients infected with RSV were more likely to be younger (<5 years of age) when compared to those infected with influenza virus. A higher hospitalization burden due to RSV compared with influenza virus among children aged <5 years has been described globally^2^, ^5^, ^8^ as well as in other African settings.^3^, ^4^, ^7^, ^10^

For both pathogens, the highest estimated hospitalization rates were observed in the Analamanga, Alaotra‐Mangoro, Atsinanana, and Haute Matsiatra Region, and the lowest in the Melaky region. Nonetheless, similar to South African, Kenyan, Rwandan, and Zambian studies,^12^, ^13^, ^20^, ^21^ no difference (with overlapping CIs) in the provincial rates of influenza‐associated SARI hospitalizations was observed, suggesting that geographical variations within countries may not affect substantially the burden associated with influenza virus or RSV infection. On the contrary, year‐to‐year variations were observed for both pathogens. This may be due to different circulating strains and patterns from year to year as well as a lower accuracy of annual estimates due to the small number of patients enrolled in a given year. This highlights the importance of estimating burden associated with influenza virus and RSV infection over several years.

Our study has limitations that warrant discussion. First, whereas we attempted to account for potential geographical differences in rates by adjusting the base rate by the regional‐level prevalence of known risk factors for pneumonia and health seeking behavior, such approach may not have accounted for the full spectrum of potential variability. Second, the completeness of the hospital records used for the retrospective record review could not be verified. An underestimation of the hospitalization rates could have occurred if not all admitted patients were recorded in the hospital books. Third, we estimated the burden of influenza‐ and RSV‐associated hospitalization only among patients hospitalized with SARI. Influenza virus infection has been reported also among patients hospitalized for respiratory illness that do not meet the SARI case definition. Specifically in a study conducted in South Africa, 5.8% of inpatients with respiratory illnesses that did not meet the SARI case definition tested positive for influenza.^36^ In addition, ecological studies have suggested that influenza and RSV are responsible for hospitalizations and deaths also among patients presenting with circulatory illnesses or other non‐respiratory and non‐circulatory syndromes.^24^, ^26^, ^27^ Lastly, individuals that may have developed influenza‐ or RSV‐associated severe illness, but did not seek care, would have been missed in our study; hence, our estimates should be considered minimum estimates.

In conclusion, we reported a substantial hospitalization burden associated with influenza virus and RSV infection especially in children aged <5 years. The Madagascar Ministry of Health has not yet implemented a national influenza vaccination program. The information presented here could be used by policymakers to consider vaccine introduction. Should an influenza vaccination program be introduced in Madagascar young children, and older adults may benefit most from annual influenza immunization. No influenza vaccine is licensed for children aged <6 months, but this group may be protected through the vaccination of their mothers during pregnancy.^37^, ^38^ The burden of RSV‐associated SARI hospitalization was higher than those of influenza. Should a RSV vaccine become available it would have the potential to prevent a substantial number of severe illnesses, especially in children.

## CONFLICT OF INTERESTS

All authors declare that they have no commercial or other associations that may pose a conflict of interest.

## AUTHOR CONTRIBUTIONS

All authors take responsibility for the integrity of the data and the accuracy of the data analysis. JR, ST, JG, MR, and JMH involved in study concept and design. JR, ST, AH, JG, and NR performed acquisition, analysis, or interpretation of data. JR, ST, and JMH drafted the manuscript. ST, AH, JG, NR, MR, and JMH critically revised the manuscript for important intellectual content.

## DISCLAIMER

The findings and conclusions in this report are those of the authors and do not necessarily represent the official position of the US Centers for Disease Control and Prevention, the World Health Organization, the Ministry of Public Health of the Republic of Madagascar, and the Institute Pasteur de Madagascar.
