# Supplementary material for: Burden and epidemiology of influenza‐ and respiratory syncytial virus‐associated severe acute respiratory illness hospitalization in Madagascar, 2011‐2016
Source: Influenza Other Respir Viruses. 2018 Dec 27;13(2):138–47. doi: 10.1111/irv.12557 (PMC6379640; doi:10.1111/irv.12557)
Supplement: Supplementary file 1 [file IRV-13-138-s001.docx]

**SUPPLEMENTAL MATERIALS**

**METHODS**

**Equations used to estimate the regional numbers and rates of influenza-and RSV-associated severe acute respiratory illness (SARI) hospitalization**

***Equation 1:*** *SARI hospitalization rates in the base region (Analamanga Region)*

$${RS}_{H,B}=\frac{{SARI}_{H,B}}{{Pop}_{B}}$$

Where:

${RS}_{H,B}$ = Base rate of hospitalized SARI (obtained from Antananarivo Renivohitra District and used as a proxy for Analamanga Region)

${SARI}_{H,B}$ = Number of total cases meeting the SARI case definition hospitalized at the 5 selected hospitals in Antananarivo Renivohitra District (data source 1).

${Pop}_{B}$ = Population of Antananarivo Renivohitra District (data source 4)

***Equation 2.a:*** *Calculation of adjustment for risk factors at the regional level for base SARI hospitalization rates*

$${Adj}_{Y}=\left( 1+\sum_{i} \left( P_{i,Y}-P_{i,B} \right)\times\left( {RR}_{i}-1 \right) \right)$$

Where:

${Adj}_{Y}$ = Adjustment factor for risk factors for SARI for region Y

$P_{i,Y}$ = Prevalence of risk factor *i* in region Y (from DHS – data source 3)

$P_{i,B}$= Prevalence of risk factor *i* in base region (from DHS – data source 3)

${RR}_{i}$ = Relative risk of SARI due to risk factor *I* (from published literature)

***Equation 2.b:*** *SARI hospitalization rates in the given region after adjustment for risk factors and healthcare-seeking behavior*

$${RS}_{H,Y}={RS}_{H,B} \times{Adj}_{Y} \times\frac{{DHS}_{H,Y}}{{DHS}_{H,B}}$$

Where:

${RS}_{H,Y}$ = Rate of hospitalized SARI in region Y

${DHS}_{H,Y}$ = Proportion of ARI cases seeking care in region Y (from DHS – data source 3)

${DHS}_{H,B}$ = Proportion of ARI cases seeking care in base region (from DHS – data source 3)

***Equation 3:*** *Influenza- and RSV-associated SARI hospitalization rates in all regions*

$${RI}_{H,Y}={RS}_{H,Y} \times I$$

Where:

${RI}_{H,Y}$= Rate of hospitalized influenza-associated SARI in region Y (including base region)

$I$ = Proportion of hospitalized SARI cases testing positive for influenza (data source 2)

$${RRSV}_{H,Y}={RS}_{H,Y} \times{RSV}$$

Where:

${RRSV}_{H,Y}$= Rate of hospitalized RSV-associated SARI in region Y (including base region)

$RSV$ = Proportion of hospitalized SARI cases testing positive for RSV (data source 2)

***Equation 4:*** *Number of influenza- and RSV-associated SARI hospitalizations in all regions*

$${NI}_{H,Y}={RI}_{H,Y} \times P{op}_{Y}$$

Where:

${NI}_{H,Y}$= Number of hospitalized influenza-associated SARI cases in region Y (including base region)

$P{op}_{Y}$= Population in region Y (including base region – data source 4)

$${NRSV}_{H,Y}={RRSV}_{H,Y} \times P{op}_{Y}$$

Where:

${NS}_{H,Y}$= Number of hospitalized RSV-associated SARI cases in region Y (including base region)

$P{op}_{Y}$= Population in region Y (including base region – data source 4)

**RESULTS**

**Table S1: Estimated mean annual number and rate of influenza- and respiratory syncytial virus-associated severe acute respiratory illness hospitalization by age group and province, Madagascar, 2011-2016.**

| **Age group (in years)** | **Influenza-associated SARI hospitalization** | | **RSV-associated SARI hospitalization** | |
| --- | --- | --- | --- | --- |
|  | **Number (95% CI)** | **Rate (95% CI)^a^** | **Number (95% CI)** | **Rate (95% CI) ^a^** |
| **Antananarivo** | | | | |
| <5 | 1,416 (1,204-1,610) | 138.3 (117.6-157.2) | 3,581 (3,300-3,854) | 349.8 (322.4-376.5) |
| 5-24 | 310 (239-382) | 10.2 (7.8-12.5) | 92 (49-140) | 3.0 (1.6-4.6) |
| 25-44 | 129 (93-172) | 8.5 (6.1-11.3) | 22 (7-43) | 1.4 (0.5-2.8) |
| 45-64 | 149 (112-185) | 22.3 (16.7-27.6) | 27 (12-44) | 4.0 (1.7-6.6) |
| ≥65 | 110 (73-149) | 61.0 (40.5-82.7) | 12 (0-28) | 6.7 (0.0-15.5) |
| ≥5 | 698 (517-888) | 12.9 (9.5-16.4) | 153 (67-255) | 2.8 (1.2-4.7) |
| All | 2,114 (1,721-2,497) | 32.8 (26.7-38.8) | 3,734 (3,367-4,109) | 58.0 (52.3-63.8) |
| **Antsiranana** | | | | |
| <5 | 251 (213-286) | 92.5 (78.3-105.2) | 633 (577-686) | 233.3 (212.7-252.9) |
| 5-24 | 54 (41-66) | 6.7 (5.1-8.2) | 16 (8-24) | 2.0 (1.0-3.0) |
| 25-44 | 23 (16-30) | 5.7 (4-7.5) | 4 (1-8) | 1.0 (0.2-1.9) |
| 45-64 | 26 (20-32) | 14.7 (11.3-18.0) | 5 (2-8) | 2.8 (1.1-4.5) |
| ≥65 | 19 (13-26) | 39.8 (27.2-54.4) | 2 (0-5) | 4.2 (0.0-10.5) |
| ≥5 | 122 (90-154) | 8.5 (6.3-10.7) | 27 (11-45) | 1.9 (0.8-3.1) |
| All | 373 (303-440) | 21.9 (17.7-25.8) | 660 (588-731) | 38.7 (34.5-42.8) |
| **Fianarantsoa** | | | | |
| <5 | 937 (797-1073) | 127.8 (108.7-146.4) | 2,372 (2,165-2,568) | 323.6 (295.4-350.3) |
| 5-24 | 188 (144-234) | 8.6 (6.6-10.7) | 55 (29-86) | 2.5 (1.3-3.9) |
| 25-44 | 79 (57-107) | 7.3 (5.2-9.8) | 15 (4-27) | 1.4 (0.4-2.5) |
| 45-64 | 91 (69-113) | 19.0 (14.4-23.6) | 15 (6-27) | 3.1 (1.3-5.6) |
| ≥65 | 67 (44-92) | 51.9 (34.1-71.3) | 7 (0-16) | 5.4 (0.0-12.0) |
| ≥5 | 425 (314-546) | 11.0 (8.1-14.1) | 92 (39-155) | 2.4 (1.0-4.0) |
| All | 1,362 (1,111-1,619) | 29.5 (24.1-35.1) | 2,464 (2,204-2,723) | 53.4 (47.8-59.1) |
| **Mahajanga** | | | | |
| <5 | 479 (406-548) | 112.9 (95.7-129.0) | 1,210 (1,104-1,313) | 285.1 (260.1-309.2) |
| 5-24 | 113 (88-140) | 8.9 (7.0-11.1) | 34 (18-52) | 2.7 (1.4-4.1) |
| 25-44 | 47 (34-63) | 7.5 (5.4-10.0) | 8 (3-15) | 1.3 (0.5-2.4) |
| 45-64 | 55 (42-68) | 19.8 (14.9-24.5) | 9 (3-16) | 3.2 (1.1-5.8) |
| ≥65 | 41 (27-55) | 54.9 (36.1-73.6) | 3 (0-10) | 4.0 (0.0-13.4) |
| ≥5 | 256 (191-326) | 11.4 (8.5-14.5) | 54 (24-93) | 2.4 (1.1-4.1) |
| All | 735 (597-874) | 27.5 (22.3-32.7) | 1,264 (1,128-1,406) | 47.4 (42.3-52.7) |
| **Toamasina** | | | | |
| <5 | 717 (610-822) | 139.5 (118.6-159.9) | 1,814 (1,658-1,967) | 352.9 (322.5-382.6) |
| 5-24 | 140 (108-174) | 9.2 (7.1-11.4) | 42 (21-64) | 2.7 (1.4-4.2) |
| 25-44 | 58 (42-79) | 7.6 (5.5-10.4) | 10 (4-19) | 1.3 (0.5-2.5) |
| 45-64 | 67 (52-85) | 19.9 (15.5-25.3) | 12 (6-21) | 3.6 (1.8-6.2) |
| ≥65 | 51 (33-67) | 56.3 (36.5-74.0) | 6 (0-12) | 6.6 (0.0-13.3) |
| ≥5 | 316 (235-405) | 11.6 (8.6-14.9) | 70 (31-116) | 2.6 (1.1-4.3) |
| All | 1,033 (845-1,227) | 32.0 (26.1-38.0) | 1,884 (1,689-2,083) | 58.3 (52.2-64.4) |
| **Toliara** | | | | |
| <5 | 668 (567-764) | 124.9 (106.0-142.8) | 1,689 (1,546-1,828) | 315.7 (289.0-341.6) |
| 5-24 | 144 (110-179) | 9.0 (6.9-11.2) | 43 (22-66) | 2.7 (1.4-4.1) |
| 25-44 | 59 (44-80) | 7.4 (5.5-10.1) | 11 (4-20) | 1.4 (0.5-2.5) |
| 45-64 | 70 (52-87) | 20.0 (14.7-24.9) | 13 (5-21) | 3.7 (1.4-6.0) |
| ≥65 | 51 (34-70) | 54.1 (36.1-74.3) | 6 (0-13) | 6.4 (0.0-13.8) |
| ≥5 | 324 (239-416) | 11.5 (8.4-14.7) | 73 (31-120) | 2.6 (1.1-4.2) |
| All | 992 (806-1,180) | 29.5 (24.0-35.1) | 1,762 (1,577-1,947) | 52.4 (46.9-57.9) |

Abbreviations: SARI: severe acute respiratory illness; CI: confidence intervals; RSV: respiratory syncytial virus.

^a^ Rates expressed per 100,000 population.


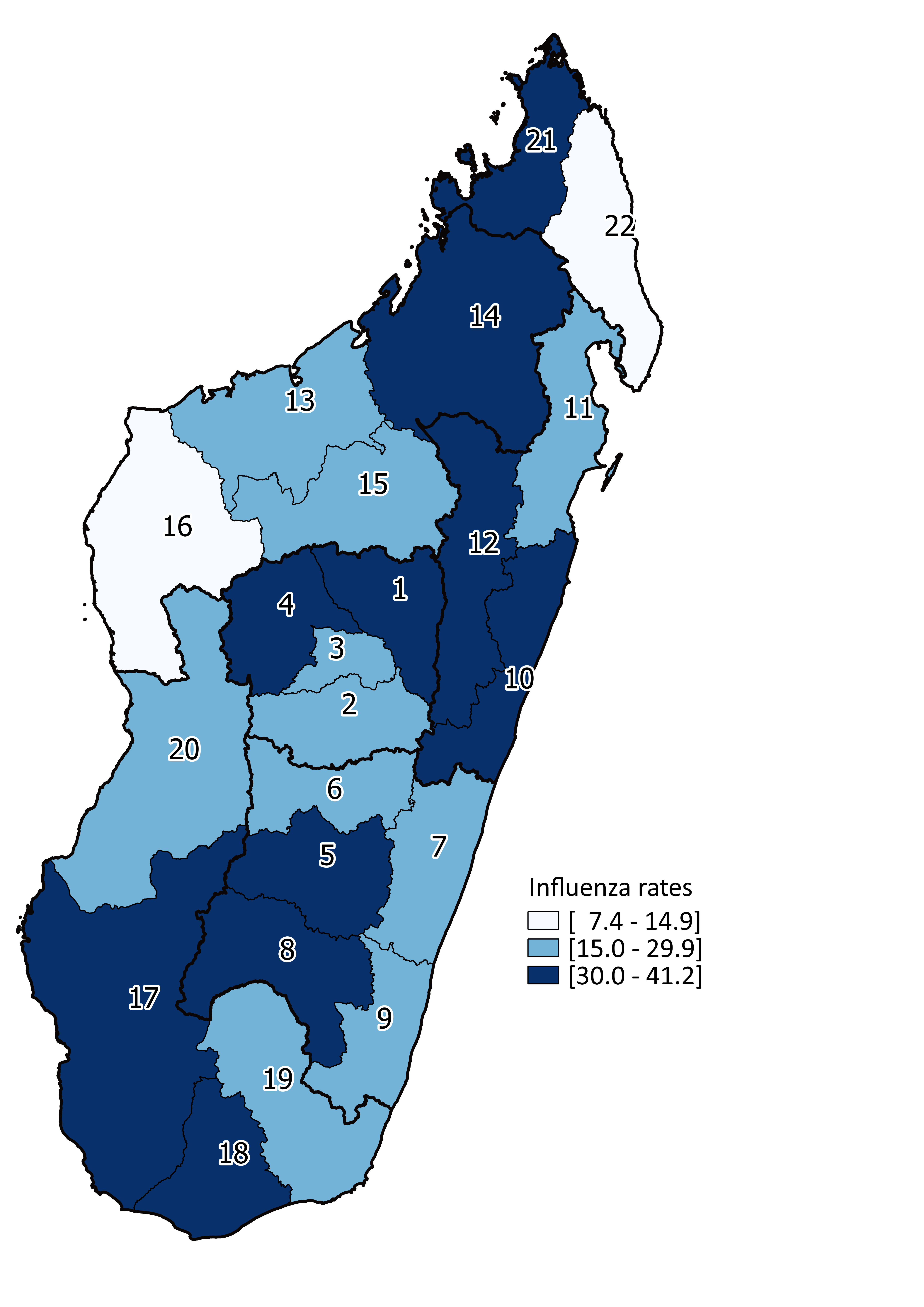

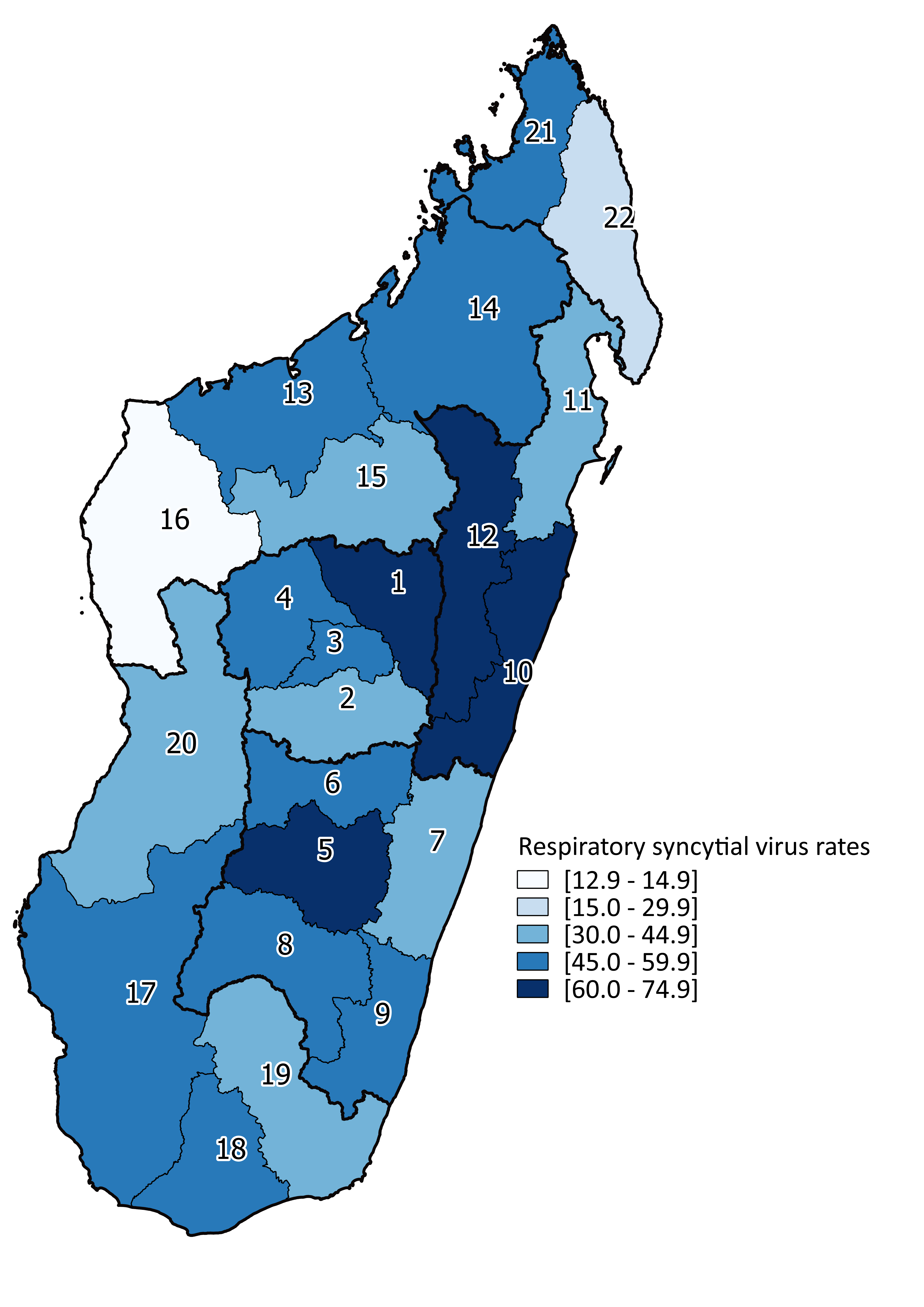


Figure S1: Estimated mean annual influenza- and respiratory syncytial virus-associated severe acute respiratory illness hospitalization rates (per 100,000 population) by region among patients of any age, Madagascar, 2011-2016. A: Influenza virus; B: Respiratory syncytial virus. Antananarivo Province: 1: Analamanga, 2: Vakinankaratra, 3: Itasy, 4: Bongolava; Fianarantsoa Province: 5: Haute Matsiatra, 6: Amoron’i Mania, 7: Vatovavy Fitovinany, 8: Ihorombe, 17: Atsimo Atsinanana; Toamasina Province: 10: Atsinanana, 11: Analanjirofo, 12: Alaotra Mangoro; Mahajanga Province: 13: Boeny, 14: Sofia, 15: Betsiboka, 16: Melaky; Toliara Province: 18: Androy, 19: Anosy, 20: Menabe, 9: Atsimo Andrefana; Antsiranana Province: 21: Diana, 22 Sava.
