# Supplementary figures and images for: Burden and epidemiology of influenza‐ and respiratory syncytial virus‐associated severe acute respiratory illness hospitalization in Madagascar, 2011‐2016
Source: Influenza Other Respir Viruses. 2018 Dec 27;13(2):138–47. doi: 10.1111/irv.12557 (PMC6379640; doi:10.1111/irv.12557)

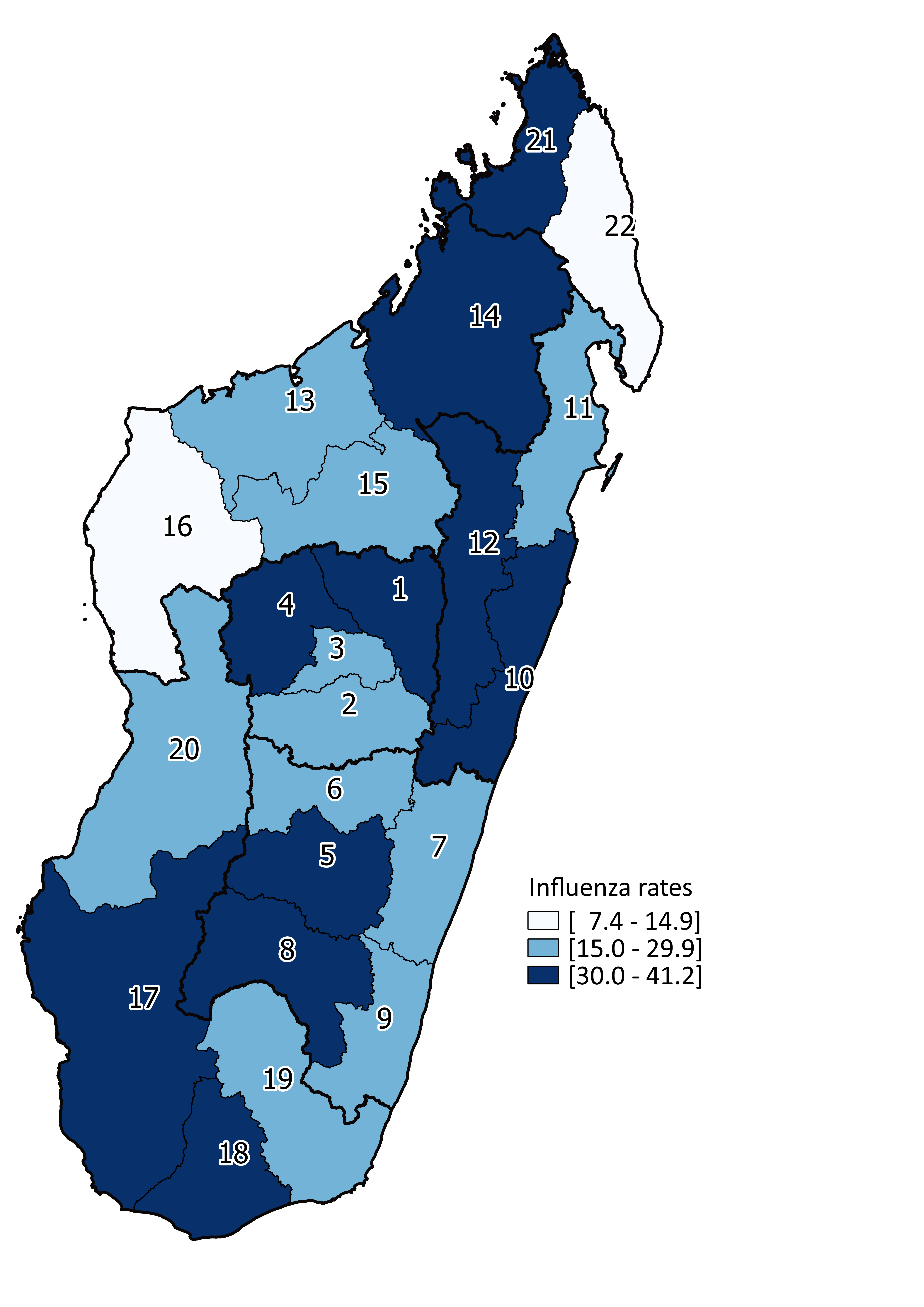

Supplement: Supplementary file 2 [file IRV-13-138-s002.tif]

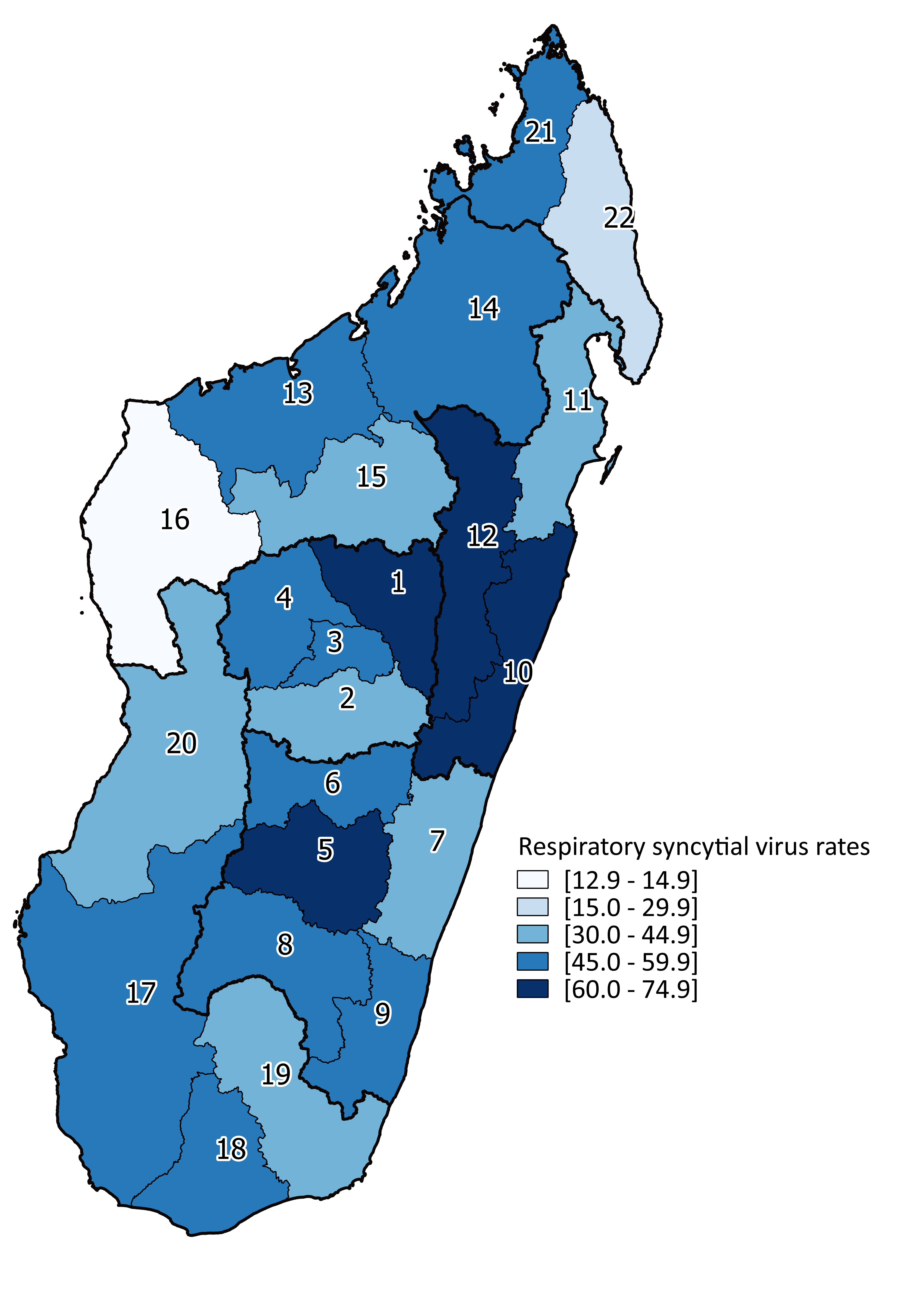

Supplement: Supplementary file 3 [file IRV-13-138-s003.tif]
